# Shared trauma reality in war: Mental health therapists’ experience

**DOI:** 10.1371/journal.pone.0191949

**Published:** 2018-02-06

**Authors:** Sara A. Freedman, Rivka Tuval Mashiach

**Affiliations:** 1 School of Social Work, Bar Ilan University, Ramat Gan, Israel; 2 Dept. of Psychology, Bar Ilan University, Ramat Gan, Israel; Harvard Medical School, UNITED STATES

## Abstract

**Introduction:**

Shared traumatic reality occurs when therapists are doubly exposed to a traumatic event, both through their clients’ experience, along with their own direct exposure. Studies have shown that a shared traumatic reality can lead to both positive and negative outcomes for therapists. Most studies have examined these reactions sometime after the end of the traumatic event, and less is known about reactions that occur during a traumatic event. In addition, most studies have assumed, rather than examined, indirect exposure. In this study, we extend this literature by examining direct and indirect exposure of therapists during a war situation, and their psychological reactions.

**Method:**

Over a period of two months in 2014, 70% of the Israeli population was exposed to rocket fire. Geographical areas differed in terms of amount of exposure, and its potential danger. 151 therapists living throughout Israel were assessed via an Internet based survey in the middle of the war, and were assessed for the effects on their professional and personal lives, degree of burnout, ways of coping and symptoms levels of PTSD and psychological distress.

**Results:**

These indicate that significant differences in direct exposure occurred depending on place of residence. PTSD levels were related to higher direct exposure, as well as prior trauma exposure, but not to indirect exposure. Indirect exposure, as measured by increased workload, was related to increased distress and emotional exhaustion.

**Discussion:**

These data shed light on the effects of direct and indirect exposure to a shared traumatic experience of war amongst therapists. The data support previous studies showing a greater effect of direct exposure on PTSD. Since indirect exposure appears to negatively impact burnout and psychological distress, rather than PTSD, this study shows that symptoms other than PTSD should be the result of in a shared traumatic reality.

## Introduction

Exposure to a traumatic event is a pre-requisite for developing posttraumatic stress disorder (PTSD, [1]). Most commonly, this exposure is direct exposure, such that the individual themselves experiences the traumatic event. Exposure can also be indirect, and a large body of studies has examined the effects of indirect exposure, for example, therapists who vicariously experience the traumatic events of their patients. Indirect exposure can lead to secondary traumatic stress: the symptoms of PTSD are present, but are considered secondary because the exposure was indirect [2]. The current definition of a traumatic event in PTSD in DSM5 has merged direct and indirect exposure, making work related indirect exposure a potentially traumatic event [3].

Shared traumatic reality describes an unusual situation, where therapists and their patients are simultaneously exposed to the same traumatic event [4]. In shared traumatic realities, direct and indirect exposure are concurrently experienced: the therapist directly experiences the traumatic event, and contemporaneously treats patients who have themselves been exposed to the same event. This has been coined double exposure[5], and typically arises in traumatic events that potentially affect whole communities, such as natural disasters and war. In order for a situation to meet definition as a shared traumatic reality, four conditions need to be met [5]: (1) The event can potentially traumatize the entire community to which the therapist and patient belong. (2) The reality shared refers to a current event, not one in the distant past. (3) Both the client and therapist belong to the community. (4) The helping professional suffers double exposure both as an individual member of the stricken community and as a professional providing services and care to persons who are themselves adversely affected by the disaster.

Shared traumatic reality has been described in a number of studies, dating from the Second World War [6]. Qualitative studies examining shared traumatic realties have documented therapists' subjective feelings of emotional and physical distress [7,8], as well as a subjective sense of compromised professional competence [9]. Studies using quantitative measures have not confirmed these findings [10]. For example, [11] [11], found that the level of distress in hospital social workers who provided emergency treatment to victims after terrorist attacks in Israel, was significantly lower than those reflected in the general Israeli population. It may be that this finding is due to a higher level of initial resilience in the social workers, compared to the general population. In addition, sharing the traumatic experience with clients may have positive aspects as well. Such effects may include a higher sense of identification and better contact with client's needs [9], a heightened commitment to the profession [12] and personal gains such as growth and satisfaction [10] This may be due to the concurrent "common fate" between therapists and their clients.

A recent study looking at double exposure has examined this in more depth amongst social workers who lived and worked during a war; this study found that double exposure was associated with both positive and negative outcomes six months after the war had ended[5] Similar results were also found amongst mental health therapists living and working in Israel [7] and in the US, following Hurricane Katrina [13].

Few studies have examined the impact of the shared trauma on the therapists’ clients. One study, following 9/11, reported that increased distress was found in only a third of clients[14]. A number of studies have shown that the level of distress experienced by the therapist is related to a greater level of prior trauma exposure [15,16]

While these studies have increased our understanding of therapists’ reactions to double exposure, they have all been carried out a period of time after the traumatic event took place, relying on retrospective data. It is important to capture the immediate effects double exposure might have, thus allowing for more accurate information regarding therapists’ burden at such times. Previous studies have rarely assessed indirect exposure, rather there has been assumption that therapists working during a traumatic event are automatically exposed indirectly [17]. In addition, the relative effects of direct versus indirect exposure have not been examined, nor the impact of the therapists’ trauma history. The current study examines immediate reactions of therapists during a war situation, considering the relative effects of direct and indirect exposure.

### Operation protective edge

There have been several military conflicts between Israel and her neighbours, and these include internal and external conflicts with Palestinians. Even between conflicts, there are often extended periods of tension and sporadic violence. This study concentrates on the military conflict that occurred in the summer, 2014, between Israel and Gaza, lasting 50 days. During this time, 3,659 rocket and mortar impact sites were found in Israel; 224 of these hit built-up areas. A further 735 were shot down by the Iron Dome missile defence system.

During the war 70 people were killed on the Israeli side, including 64 soldiers, and more than 2,100 people were killed in Gaza Strip in the fighting and many thousands more were wounded [18].

For civilians living in Israel during the conflict, there were specific stressors that had not been experienced by much of the population in previous conflicts. For instance, rocket sirens went off regularly, and required citizens to run to a secure room and remain there for ten minutes. The number of sirens varied by area, with citizens in the south exposed to a greatly increased number. In addition, the amount of time that people had to get to a secure room before a rocket struck varied between 15 seconds (in areas in the South) to 90 seconds (in the North of Israel). Some areas (such as the south of the country and the north) had much more previous experience with rocket attacks from other conflicts; citizens living in the center of the country had rarely experienced such attacks. Moreover, many families had members who were involved in the combat service in fighting units, inside Gaza; this was an additional stressor. The “home front” was under constant attack during the conflict, although daily life continued, with work places remaining on normal hours.

This study examined a diverse population of therapists, including social workers, psychologists and psychiatrists, with differing levels of direct and indirect exposure to this war situation. Outcome measures were symptom levels of PTSD, general levels of distress, and burnout. Higher levels of PTSD, distress and dysfunction were hypothesized to be related to greater direct and indirect exposure to war. Burnout, as well as personal accomplishment, were hypothesized to be related to greater indirect exposure. In addition, higher levels of prior trauma exposure were hypothesized to be related to higher levels of current PTSD symptoms. Coping strategies that have been identified as less adaptive (self-distraction, denial, substance use, disengagement, venting and self-blame) were hypothesized to be related to higher symptoms levels, and more burnout.

## Method

### Participants

One hundred and fifty-one people participated in the study, 121 women (80%) and 28 men (18.5%). All participants worked in the therapy professions: 30 (20.8%) were social workers, 87 (60.4%) were psychologists, 5 (3.5%) were psychiatrists and 22 (15.3%) were classified as other ((e.g. social work and psychology interns, counsellors)). Twenty therapists lived in the south, 35 in the Jerusalem area, 85 in the centre of the country, and 12 in the north.

### Measures

The following measures were used:

Demographic questionnaire: this included background data, such as age, gender, occupation, and marital status. Previous traumatic experience was assessed using the LEC [19], which lists 14 different potentially traumatic events. Participants indicate whether they were exposed directly, or in a work-related context. A total number of events experienced was calculated for directly experienced (personal trauma) and work-related events (work trauma).

#### Effects of war

Disruption of Daily Routines: this 9-item questionnaire assessed how much participants’ daily lives had been affected by the conflict. It has been used in previous studies [20]. Each item is marked on a 5-point severity scale (0 = no disruption; 4 = extreme disruption). The total disruption score is calculated by summing all items. The instruments consistency was satisfactory (Cronbach’s alpha = 0.85).

Direct Exposure to the war was assessed by four questions. Firstly, participant’s residence was divided into one of two regions (*South*, *rest of country*). Residents of the south of the country had been exposed to a longer-term risk with many months of rocker fire preceding the current escalation. During the current war, residents risk was objectively higher, since they had less time to reach cover during rocket attacks. Secondly, participants indicated to how many rocket attacks they had been exposed. Thirdly, participants indicated whether a family member was serving as a combat soldier. Lastly, participants were asked whether they needed to relocate as a result of the war.

Indirect exposure to the war was assessed by three sections. Firstly, two dichotomous questions assessed whether the therapist had treated acute stress disorder as a result of the war (TreatASD), and whether they had treated a soldier (TreatSoldier). Secondly, each therapist gave information regarding the exposure of up to five of their patients: had the patients been exposed to sirens, or a worsening of their symptoms, giving a score of 0 (no patients exposed to sirens / showed worsening of symptoms) to 5 (all patients exposed to sirens / showed worsening of symptoms). Lastly, workload was assessed by three items, marked on a 5-point scale. These assessed increased number of hours due to the war, increased number of patients, and working harder.

#### Symptom measures

*Psychological Distress*, *K-6* [21] is a short, six question screening scale examining depression and anxiety. It was developed from a previous ten items instrument (the K10) for the US National Health Interview Survey. Both the K10 and K6 have consistent psychometric properties across major socio-demographic sub-samples. They strongly discriminate between community cases and non-cases of DSM-IV/SCID disorders.

*Burnout*: Maslach Burnout Questionnaire [22] is a 22 item questionnaire assessing three subscales–emotional exhaustion, depersonalization and personal accomplishment. For the purposes of this study, the abbreviated version (9 items) was used [23]. High scores on the first two subscales, and a low score on the last one, indicate high levels of burnout. In this study, Cronbach’s alpha for the emotional exhaustion subscale was 0.8, depersonalization 0.8 and personal accomplishment 0.7.

*PTSD Symptoms*, *PCL-5* [24] is a 20-item self-report questionnaire, assessing PTSD symptoms according to DSM 5 [25]. This version was translated into Hebrew and back translated to ensure accuracy. Cronbach’s alpha for this study was 0.935.

*Functionional impairment*: subjects’ level of functioing was assessed using four questions [20]: ability to pursue task performacne, controllability of emotions, ability to sustain rewarding interpersonal contacts and positive self-value. All items were assessed on a 5-point Likert scale, with higher scores indicating higher functioing.

*Brief COPE* [26] is a 28 item self-report questionnaire that assesses subjects’ coping strategies. Each item is scored on a 4-point scale, and 14 sub-scales are calculated: self-distraction, active coping, denial, substance use, use of emotional support, use of instrumental support, behavioral disengagement, venting, positive reframing, planning, humor, acceptance, religion, and self-blame.

### Procedure

This study was an internet based study, including 151 therapists living in Israel during the conflict during July-August, 2014. The study used Qualtrics software to collect data. The authors sent an email with a link to the study questionnaires to colleagues, mailing lists for therapists, and websites for therapy related organizations. Individuals were asked to pass the link on to any therapist in the country. Completion was anonymous (and therefore did not require signed informed consent), and the study received ethical approval from the School of Social Work, Bar Ilan University. Data collection took place between the third and fifth weeks of the conflict.

### Data analysis

Statistical analyses were performed using SPSS software, version No. 21. Differences between groups were calculated using t and χ^2^ tests. Relationships between the study variables were calculated using Pearson and Spearman correlations. The relative contribution of each variable to explain the variances of outcome variables were calculated using one-way ANOVA. An a prior statistical power analysis was performed for sample size estimation. The effect size (ES) in this study was considered to be medium using Cohen's criteria[27]. With an alpha = .05 and power = 0.90, the projected sample size needed with this effect size (GPower 3.1[28]) is approximately N = 130 for regression analysis.

## Results

Table 1 describes background characteristics of the sample. There were no significant differences in terms of any of these variables, between therapists living in the South, compared with those living in the rest of the country.

**Table 1:** Demographic characteristic of residents of the south and the rest of the country.

Char	Rest of Country (N = 131)		South (N = 20)		Test
	Mean/N	SD/%	Mean/N	SD/%	
**Age**					M-W U = 1243.50, ns
18–35	56	42.7	7	35	
36–45	35	26.7	7	35	
46+	40	30.5	6	30	
**No Children**	2.57	1.43	2.83	1.38	t(111) = 0.96, ns
**Gender**					X^2^(1) = 1.17, ns
Male	26	20.2	2	10	
Female	103	79.8	18	90	
**Marital Status**					X^2^(2) = 2.96, ns
Single	17	13	0	0	
Married	95	72.5	17	85	
Divorced*	19	14.5	3	15	
**SES**					M-W U = 1188.50, ns
Below average	35	26.7	5	25	
Average	32	24.4	8	40	
Above average	64	48.9	7	35	
**Years work**					M-W U = 1183.50,ns
<2	25	19.4	3	15	
2–5	21	16.3	4	20	
6–10	25	19.4	2	10	
10+	58	45	11	55	
**Profession**					X^2^ (2) = 0.09, ns
Social work	26	20.5	4	23.5	
Psychologist	77	60.6	10	58.8	
Other	24	18.9	3	17.6	

Participant age and years of work were both correlated to personal accomplishment (age: r = -.36, p < .001; work: r = -.36, p < .001), emotional exhaustion (age: r = -.17, p < .05; work: r = -.18, p < .05) and dysfunction (age: r = .20, p < .05; work: r = .19, p < .05(. Thus, older and more experienced participants reported lower levels of personal accomplishment and emotional exhaustion, and higher levels of dysfunction.

Socioeconomic status was also negatively correlated to personal accomplishment (r = -.27, p = .001). Thus, higher SES was related to lower levels of accomplishment.

Children number was correlated to the K6 score (r = -.27, p < .005) and dysfunction (r = .24, p < .05). Participants with more children had lower K6 scores but reported higher levels of dysfunction.

Marital status had an impact on the PTSD symptoms measure (F(2,122) = 4.52, p < .05, η^2^ = .069). Bonferroni post-hoc tests showed that mean symptom levels among divorced participants (M = 19.50, SD = 18.04) was significantly higher compared to married participants (M = 10.79, SD = 10.20, p < .05). Symptom levels among single participants did not differ from both other groups (M = 10.73, SD = 11.25).

Finally, therapists' profession had an impact on personal accomplishment score (F (2,134) = 3.17, p < .05, η^2^ = .045). Bonferroni post-hoc test showed that mean personal accomplishment among psychologists (M = 1.65, SD = 1.21) was significantly higher compared to the "others" group (M = 1.01, SD = 0.70, p < .05). Personal accomplishment level among social workers did not differ from both other groups (M = 1.44, SD = 1.20).

Participants' gender did not affect any of the symptom measures.

Table 2 shows statistics regarding exposure and disruption. In terms of direct exposure, therapists from the south reported significantly higher levels of all variables, with the exception of a soldier in the family. Similarly, therapists in the south reported significantly higher levels of disruption. No significant differences were found regarding the indirect exposure.

**Table 2:** Exposure and disruption.

	Rest of country N = 131	South N = 20	Analysis
	N (%)	N (%)	χ^2^
**Direct Exposure**			
House Relocation	0	6 (37.5%)	45.6***
Soldier in family	3 (2.6%)	2 (13.3%)	4.2, ns
**Indirect Exposure**			
Treated ASD	63 (52.9%)	10 (58.8%)	0.2, ns
Treated Soldiers	13 (10.9%0	1 (6.3%)	0.3, ns
	**Mean (SD)**	**Mean (SD)**	**F**
**Direct Exposure**			
Rocket Attacks	1.5 (0.8)	2.9 (0.7)	37.5 ***
**Indirect Exposure**			
Patient/s exposed to sirens	1.7 (2.2)	1.0 (1.9)	2.0, ns
Patient/s got worse	0.8 (1.3)	0.6 (1.2)	0.4, ns
**Workload**			
More hours work	1.72 (0.9)	2.43(1.5)	8.8**
More patients	1.6 (0.9)	2.29 (1.5)	7.2**
Work harder	1.8 (0.9)	2.6 (1.6)	8.7**
Total Workload	2.0 (4.3)	5.1 (2.4)	8.4**
**Disruption**			
Work	1.98 (0.9)	3.5 (1.1)	39.8***
Leisure activities	2.6 (0.9)	4.2 (0.7)	47.1***
Rel’ships with friends residing elsewhere	2.1 (1.1)	3.3 (1.4)	19.6***
Relationships with family residing elsewhere	1.2 (0.5)	2.1 (1.5)	25.7***
Relationships with immediate family	2.06 (1.4)	1.39 (0.7)	9.7***
Financial burden	1.52 (0.7)	2.47 (1.3)	17.8***
Day-to-day schedule	1.98 (0.9)	4.0 (0.8)	73.8***
Mobility	1.67 (0.9)	2.8 (1.2)	23.0***
Overall qol	2.5 (0.8)	3.4 (1.0)	18.7***
Total disruption score	14.4 (8.4)	23.5 (12.6)	18.1***

***p < .001

**p < .01.

ASD- Acute Stress Disorder; QOL–quality of life

As can be seen in Table 3, there were no significant differences in symptom levels between residents of different areas, with the exception of PTSD symptoms score (t(123) = 2.50, p < .05), which was higher among therapists from the south compared to therapists from the rest of the country.

**Table 3:** Symptom levels.

	Rest of Country N = 131	South N = 20	Analysis
	Mean (SD)	Mean (SD)	T
PTSD	11.23 (11.59)	19.71 (14.71)	t(123) = 2.50*
K6	12.28 (3.61)	13.50 (2.25)	t(149) = 1.37, ns
Burnout: Personal accomplishment	1.43 (1.18)	1.78 (0.68)	t(141) = 1.25, ns
Burnout: Depersonalization	0.79 (0.88)	0.89 (1.31)	t(141) = 0.67, ns
Burnout: Emotional exhaustion	2.24 (1.18)	2.67 (1.50)	t(139) = 0.16, ns
Dysfunction	8.66 (1.77)	7.80 (1.78)	t(120) = -1.77, ns

*p < .05.

Correlation tables

Bivariate correlations were carried out between the outcome variables (PTSD symptoms, dysfunction, burnout and distress) and disruption, direct and indirect exposure (Table 4), as well as past trauma and coping strategies (Table 5).

**Table 4:** Correlations between study variables.

	PCL	K6	Persacc	Depers	Emext	Dysfunct.	House Relocation	Siren amount	Family soldier	Treat ASD	Treat soldiers	Pt siren	Pt worse	Workload
PCL	-													
K6	.66***	-												
Persacc	.22*	.06	-											
Depers	.08	.13	.19*	-										
Emext	.39***	.49***	.34***	.40***	-									
Dysfunction	-5.3***	-.50***	-.42***	-.19*	-.33***	-								
House Relocation	.27***	.21*	.28**	-.06	.04	-.23*	-							
Siren amount	.34***	.17	.08	.04	-.03	-.22*	.35***	-						
Family soldier	.17	.08	.10	.04	.09	-.08	-.04	.13	-					
Treat ASD	.15	.06	-.17	-.07	.06	.10	.13	.34***	.02	-				
Treat soldiers	.07	.08	.00	.11	.23**	.09	.05	.13	-.06	.20*	-			
Pt siren	.09	.07	.15	.00	.03	-.09	.05	.17	.05	-.01	-.17	-		
Pt worse	-.02	.04	-.39*	-.26	-.09	.18	.19	.06	.20	.33*	.20	.08	-	
Workload	.12	.19*	.06	.08	.29***	.02	.17	.30**	.03	.43***	.24**	.18	.42**	-
Total disruption	.47***	.36***	-.18*	.01	.13	-.34***	.47***	.35***	.08	.15	-.20	.23	.15	.22**

*p < .05

**p < .01

***p < .001.

Persacc: personal accomplishment, Maslach Burnout Inventory; Depers: depersonalization, Maslach Burnout Inventory; Emext: Emotional Exhaustion, Maslach Burnout Inventory; Treat ASD: treat Acute Stress Disorder; Pt Siren: number of patients exposed to sirens; patient worse: number of patients reported feeling worse.

**Table 5:** Correlations between symptom measures, past trauma and coping strategies.

	Work trauma	Personal trauma	Self-distract.	Active coping	Denial	Substance	Emot. Sup.	Instru sup	Diseng	Ventilation	Positive reap.	Plan	Humour	Acceptance	Religion	Self-blame
PCL	-.15	.23**	.12	.01	.07	-.02	-.06	-.06	-.20*	-.10	-.10	-.05	.01	-.04	-.03	.04
K6	-.15	.15	.18*	.11	.17*	.10	.09	.08	-.03	.03	.00	.09	.05	-.01	-.14	.09
Persacc	-.24**	-.27***	-.05	-.12	-.11	-.14	-.10	-.10	-.29***	-.12	-.16	-.09	-.13	-.15	-.15	-.06
Depers	.04	-.07	-.06	-.09	.08	-.03	-.09	-.09	-.13	-.11	-.12	-.09	-.09	-.14	-.07	-.03
Emext	-.07	-.02	-.08	-.14	-.05	.01	-.19	-.20*	-.18*	-.26**	-.15	-.18*	-.13	-.22*	-.15	-.14
Dysfunction	.13	-.12	-.08	-.01	-.10	.07	-.02	-.01	.25**	.05	.13	.01	.03	.11	.03	-.06

*p < .05

**p < .01

***p≤.001.

Persacc: personal accomplishment, Maslach Burnout Inventory; Depers: depersonalization, Maslach Burnout Inventory; Emext: Emotional Exhaustion, Maslach Burnout Inventory, Self-distract: Self-distraction, Emot. Sup.: Emotional support, Intru. Sup.: Instrumental support, Diseng: Disengagement, Positive reap: Positive reappraisal.

### Regression analyses

Based on the previous parts' findings, five multiple regression analyses were conducted, in order to evaluate the contributions of background, exposure and coping variables to the prediction of symptom measures. Every regression analysis concerned one of the five symptom measures (PCL score, K6, personal accomplishment, emotional exhaustion and dysfunction. Depersonalization was not included in these analyses because none of the potential predictors had significant relationships with it). The predictors in each analysis were the variables which found to be related to the relevant dependent factor (age and work years were highly intercorrelated, thus, only age was entered to the relevant regressions). Therefore, in the regression analysis for the prediction of PCL score, marital status, house relocation, siren amount, disruption, personal trauma and disengagement were entered as predictors; In the regression analysis for the prediction of K6 score, children number, house relocation, workload, disruption, self-distraction and denial were entered as predictors; In the regression analysis for the prediction of personal accomplishment score, age, SES, profession, house relocation, patient worse, disruption, work trauma, personal trauma and disengagement were entered as predictors; In the regression analysis for the prediction of emotional exhaustion score, age, treat soldier, workload, instrumental support, disengagement, ventilation, planning and acceptance were entered as predictors; In the regression analysis for the prediction of dysfunction score, age, children number, disruption and disengagement were entered as predictors. Marital status and profession, which were categorical variables, were entered as dummy variables. Regressions results are presented in Table 6.

**Table 6:** Regression analyses.

DV	PCL		k6		Peracc		Emext		Dysfunction	
Predictor	B (SE)	Β	B (SE)	β	B (SE)	Β	B (SE)	β	B (SE)	β
age					-0.18 (0.18)	-.21	-0.35 (0.14)	-.24*	0.06 (0.23)	.03
Children no.			-0.74 (0.21)	-.35***					0.22 (0.13)	.19
Marital: married	-4.75 (2.98)	-.20								
Marital: divorced	0.92 (3.76)	.03								
SES					-0.20 (0.18)	-.23				
Profession: SW					-0.16 (0.34)	-.09				
Profession: Psy.					0.15 (0.30)	.10				
Relocation	6.26 (5.71)	.11	-1.65 (1.63)	-.11	1.03 (0.77)	.22				
Siren amount	0.39 (1.24)	.03								
Treat soldiers							0.67 (0.38)	.17		
Patient worse					-0.12 (0.09)	-.22				
Workload			0.25 (0.11)	.23*			0.11 (0.04)	.24*		
Disruption	0.60 (0.18)	.36***	0.17 (0.06)	.32**	-0.03 (0.02)	-.24			-0.09 (0.03)	-.31**
Personal trauma	0.67 (0.31)	.21*			-0.07 (0.05)	-.26				
Work trauma					0.01 (0.04)	.03				
Self-distraction			-0.22 (0.20)	-.16						
Denial			0.42 (0.34)	.18						
Instrumental sup.							0.15 (0.16)	.36		
Disengagement	-0.70 (0.47)	-.13			-0.07 (0.11)	-.10	-0.09 (0.08)	-.16	0.23 (0.12)	.19
Ventilation							-0.39 (0.19)	-.90*		
Planning							0.08 (0.12)	.19		
Acceptance							0.10 (0.10)	.24		
**R**^**2**^	.32, F(7,86) = 7.22***		.23, F(6,75) = 5.07***		**.24, F(10,27) = 2.16**		.16, F(8,97) = 3.54***		.16, F(4,82) = 5.23***	

*p < .05

**p < .01

***p≤.001.

Parsec: personal accomplishment, Maslach Burnout Inventory; Depers: depersonalization, Maslach Burnout Inventory; Emmet: Emotional Exhaustion, Maslach Burnout Inventory, Marital: Marital status, SES: Socio-economic status, SW: Social worker, Psy: Psychologist, Patient worse: Total number of patients reported feeling worse, Instrumental. Sup.: Instrumental support.

As can be seen in Table 6, the regression model for the prediction of the PCL score significantly explained 32% of the variance. Disruption and personal trauma had a significant contribution to the prediction. The regression model for the prediction of the K6 score significantly explained 23% of the variance. Children number, workload and disruption had a significant contribution to the prediction. The regression model for the prediction of the personal accomplishment score was not significant, although it explained 24% of the variance. None of the potential predictors had a significant contribution to the prediction. The regression model for the prediction of the emotional exhaustion score significantly explained 16% of the variance. age, workload and ventilation had a significant contribution to the prediction. Finally, the regression model for the prediction of the dysfunction score significantly explained 16% of the variance. Only disruption had a significant contribution to the prediction.

## Discussion

This study described the immediate effects of a shared traumatic reality on working mental health practitioners during a war. The results indicate that posttraumatic symptom levels were related to both direct exposure, in terms of daily disruption, and to prior exposure to traumatic events, but not to indirect exposure. This supports the literature that has found a dose effect, such that greater exposure is related to a stronger reaction [29]. It is interesting that indirect exposure did not contribute to PTSD symptoms, suggesting that direct exposure, both current and past, has a greater impact on PTSD, and these results replicate previous studies [30]. It is important to note that the highly exposed group did not show higher levels of dysfunction, and this combination of relatively higher PTSD together with low dysfunction, has been found previously in a civilian population exposed to ongoing terror [20]. Since the diagnosis of PTSD requires a change in functioning, these results probably indicate that higher levels of PTSD symptoms were not an indication of psychopathology per se, but rather normal reactions that occur because of exposure to war. For instance, thinking about the war, sleeping badly, and forced behavioral avoidance, are all natural reactions to a war situation. These results emphasize the need to take into account changes in dysfunction when assessing PTSD levels in populations that are exposed to ongoing stressful environments. The finding that disruption in daily routines, rather than other types of direct exposure, such as rocket fire, explained most of the variance in PTSD, as well as distress levels and dysfunction levels, reinforces this view. Moreover, this replicates studies that show that daily hassles are more significant stressors during a war, than other types of exposure [31]

General levels of distress, as measured by the K6, were related to both indirect exposure, specifically increased workload, and direct exposure via daily disruption, as well as being a parent to more children.

The results regarding burnout show that emotional exhaustion was related to increased workload, being older and less use of ventilation as a coping strategy. This is an intuitive finding replicating previous studies in different work situations [32]. Since emotional exhaustion is likely to have a negative effect on other areas of functioning, it is possible that reducing war related workload (perhaps by accessing volunteers) might reduce the emotional burden placed on mental health therapists during war. In addition, providing forums for safe ventilation may reduce burnout.

Shared traumatic reality is a combination of direct and indirect exposure to a traumatic event. In this study, levels of indirect exposure did not differ even in areas where the direct exposure was significantly higher. Possibly therapists did not work and live in the same area, although this seems unlikely. A different explanation is that the amount of therapeutic work increased due to the war, but that the content of sessions did not touch greatly on the effects of the war, but concentrated on other issues; since the levels of indirect exposure seem quite low, then this is plausible, and would confirm previous findings[14]. Since this is preliminary, these results need to be further verified, but they highlight the importance of assessing patients’ experiences in a shared traumatic reality, and the necessity to ascertain the content of therapy sessions.

Indirect exposure did not contribute at all to the levels of PTSD in this study, although increased workload was associated with higher levels of distress and burnout. Taken together, these results indicate that direct and indirect exposure may be related to different symptom profiles, and show the importance of assessing both types of exposure, as well as symptoms other than PTSD in a situation of shared traumatic reality.

This study was limited in five main areas. Firstly, the use of a small, convenience sample makes it difficult to know whether the results here are representative. Secondly, most subjects were women, and it is not known whether this biased the results. Thirdly, use of self-report questionnaires instead of clinical interviews does not allow diagnosis of psychopathology. Fourthly, the use of an internet based study does not allow for sampling bias to be ascertained. Lastly, a cross sectional study does not allow for measuring changes over time, which may be particularly pertinent in a war situation; additionally, participants took part in the study at different points during the war. Despite these limitations, these data give an insight into the immediate reactions of mental health professionals during a conflict. These findings may point to the need to be more aware to therapists' needs and overload in times of stress and war, and to allocate time for rest after such periods, or preparing for an efficient allocation of workload in such times.
