# Correction: Shared trauma reality in war: Mental health therapists' experience

**DOI:** 10.1371/journal.pone.0194359

**Published:** 2018-03-08

**Authors:** Sara A. Freedman, Rivka Tuval Mashiach

There is an error in the final sentence of the abstract. The correct sentence is as follows: Since indirect exposure appears to negatively impact burnout and psychological distress, rather than PTSD, this study shows that symptoms other than PTSD should be assessed in a shared traumatic reality.
